# Online Suicide Identification in the Framework of Rhetorical Structure Theory (RST)

**DOI:** 10.3390/healthcare9070847

**Published:** 2021-07-05

**Authors:** Xingyun Liu, Xiaoqian Liu

**Affiliations:** 1 Key Laboratory of Adolescent Cyberpsychology and Behavior, Ministry of Education, School of Psychology, Central China Normal University, Wuhan 430079, China; liuxingyun@ccnu.edu.cn; 2 Institute of Psychology, Chinese Academy of Sciences, Beijing 100101, China

**Keywords:** suicide identification, online posts, rhetorical structure theory (RST)

## Abstract

Background: Suicide is a serious social problem. Substantial efforts have been made to prevent suicide for many decades. The internet has become an important arena for suicide prevention and intervention. However, to the best of our knowledge, only one study has analyzed suicidal comments online from the perspective of rhetorical structure with incomplete rhetorical relations. We aimed to examine the rhetorical differences between Chinese social media users who died by suicide and those without suicidal ideation. Methods: The posts of 15 users who died by suicide and 15 not suffering from suicide ideation were annotated by five postgraduates with expertise in analyzing suicidal posts based on rhetorical structure theory (RST). Group differences were compared via a chi-square test. Results: Results showed that users who died by suicide posted significantly more posts and used more rhetorical relations. Moreover, the two groups displayed significant differences in 17 out of 23 rhetorical relations. Limitations: Because this study is largely exploratory and tentative, caution should be taken in generalizing our findings. Conclusions: Our results expand the methods of RST to the online suicidal identification field. There are implications for population-based suicide prevention by combining rhetorical structures with context analysis.

## 1. Introduction

Suicide is a serious public health problem, with global incidence trending upward over recent decades [1]. For China, the suicide rate has been decreasing because of economic growth, improved health care and other social changes [2]. However, as the most populous country in the world, China is still suffering significant losses due to suicide deaths [1]. In China, approximately 200 million people attempt suicide annually, and even more worrisome, two-thirds of those people are aged between 15 and 34 years [3]. According to the World Health Organization, suicide is the fourth leading cause of death among people aged 15 to 29 years for both sexes, and 88% of adolescents who took their own lives by suicide were from low- and middle-income countries (e.g., China) where nearly 90% of the world’s adolescents live [4]. Therefore, suicide prevention for young people is imperative in developing countries such as China. As with any suicide intervention, identification is vital because suicide can be prevented if suicidal ideation can be identified sufficiently early [5].

The Internet may bring light to this field, as it has been supported as an effective tool to identify and prevent suicide for young people [6,7,8]. Cash et al. discerned potentially suicidal themes based on content analysis theory. They found that people with suicide risk often talked about relations, mental health, substance use, suicide methods, and other themes on Myspace [9]. Liu et al. recognized social media users suffering from suicidal ideation with the assistance of suicidal identification machine learning models. They were able to improve the performance of their machine learning models because they used professional annotators, a theoretical-based feature selection strategy, and a larger dataset size. However, irrespective of the analysis method, existing research on the identification of suicidal information mainly focused on contents [7]. Johannßen and Biemann summarized research about psychology by using natural language processing and they drew the conclusion that to understand the psyche through language, it is more valuable to focus on how people express themselves rather than what they say. Apart from contents, text can also be analyzed from the view of morphology or syntax, such as discourse relations [10].

Rhetorical relation, also termed coherence relation, discourse relation, or conjunctive relation refers to the text coherence between different parts of texts in which every part of a discourse has a function to play with respect to other parts in the discourse [11], and it is a kind of structural relationship and an interpretation of the purpose and meaning of a text. It has a strong explanatory power in terms of speech intention. To the best of our knowledge, only one research study demonstrates this problem from the perspective of rhetorical relations. Kavuluru et al. used the N-gram, a linguistic inquiry and word count (LIWC), and rhetorical relations to classify helpful comments in an online subreddit community called Suicide Watch. They concluded that rhetorical relations could increase recall substantially better than other approaches [12]. However, they only used limited rhetorical relations without explanation and the sample of annotations was biased. Therefore, more research is required to analyze suicidal text from the viewpoint of text organization.

Rhetorical structure theory (RST) was built by Mann and Thompson to parse the organization of natural texts, that is, analyzing the hierarchy and relational structure of a text to demonstrate its communicative function [13]. RST is one of the most popular discourse analysis theories, and it has been widely used in linguistics, psychology, computer science, and many other fields [14]. Typically, the spans in relations are asymmetric, and a discourse can be divided into non-overlapping parts called the nucleus and the satellite. The nucleus is supposed to be the most cardinal component in a discourse that expresses the key ideas whereas the satellite is seen as a secondary part that contains supplementary information for the nucleus. Apart from constraints on the nucleus, the satellite and their combination, the theory also emphasizes the effects of different relations. For example, Evidence is one of the rhetorical relations which is often used in a text. Evidence exists when the satellite is used to increase readers’ belief of the nucleus. The following is a classical text containing an Evidence relation used by Mann and Thompson in their study: “1. Tempting as it may be, we shouldn’t embrace every popular issue that comes along. 2. When we do so, we use precious, limited resources where other players with superior resources are already doing an adequate job.” The first sentence is the nucleus, and the second sentence is the satellite. The purpose of the satellite is to increase the readers’ belief as to why we should not embrace every popular issue that comes along. There also exist multi-nuclear relations, too, such as Contrast. The definitions of all of the relations and a deeper introduction of RST can be found on Mann’s website (http://www.sfu.ca/rst, accessed on 2 July 2021).

The relations that RST provides are an open system that can be expanded according to the purpose of the analysis. The original version produced 24 relations, and this later developed into 30 relations [15]. In this paper, we used the 30-relation set, which is summarized in Table 1. More details (e.g., the differences or the constraints of each relation) can be found on Mann’s website (http://www.sfu.ca/rst, accessed on 2 July 2021).

In addition, due to the abovementioned nucleus–satellite distinction, there is a hierarchy in the analysis. RST structures can typically be illustrated as top-to-bottom trees, with higher level relations that include lower-level ones. For example, to analyze the text “I am depressed, so I will kill myself. No other important reasons, so please do not worry about my leaving. Bye.” based on RST, the first step is to divide it into different units (i.e., the minimal elements in analysis) which often consist of independent clauses, as follows:

1. I am depressed,
2. so I will kill myself.
3. No other important reasons,
4. so please do not worry about my leaving.
5. Bye.

**Figure 1:**
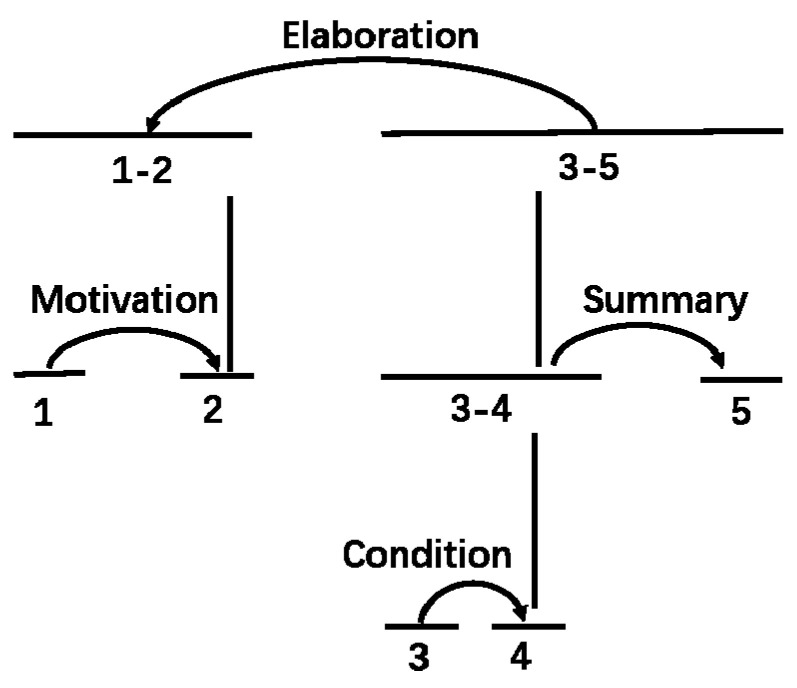
Diagram of an RST analysis.

As shown in Figure 1, the numbers delegate the units into which the text has been divided. The horizontal lines represent spans that may consist of one or multiple units. For example, Units 1–2 can be a span, as can Unit 5. The vertical lines demonstrate the hierarchy of the texts. The example text can be then decomposed into three levels, as shown. Finally, the arcs with relation names on them point from the satellites to the nuclei. The main relation of the text is Elaboration, with the nucleus composed of Units 1–2.

The main purposes of RST are to determine how the writers realize their writing intentions and how they achieve the goal of communication through the comprehensive analysis of relation structures. More importantly, the analytical results of RST are not affected by the original text size [13].

It has been demonstrated that young people have strong motivations to talk about their suicidal ideations online [7]. Existing research indicates that young people feel more comfortable when they discuss suicide-related topics on social networks than in real life [16]. In addition, some youngsters may express their suicide ideation online before they share their distress with their doctors [17]. Therefore, using RST to analyze young people’s suicidal-related texts online may have far-reaching importance for suicide identification.

In this study, to obtain a profound understanding of the online writing of people who died by suicide from the perspective of the rhetorical relations, we analyzed social media users’ (i.e., microbloggers) posts on Sina Weibo (i.e., the Chinese version of Twitter). Because previous studies rarely analyze from this perspective, we provide a broad intuition and interpretation of trends rather than tests of specific hypotheses in this research. We compared the rhetorical structure of the posts between microbloggers who died by suicide and those without suicidal ideation to determine the rhetorical relation characteristics of people who died by suicide.

## 2. Methods

### 2.1. Participants and Materials

With the assistance of the concerned people within Sina Weibo and news reports, we identified 45 microbloggers who died by suicide. Based on previous experience [18] and consultation with specialists, we set the criteria for exclusion as follows: (1) the number of self-generated posts is less than 50 (*N* = 13); (2) the length of the corpus for each user is not internally consistent (*N* = 4); (3) the number of corpus items is under 500 (*N* = 3); (4) there are dirty data in the corpus (*N* = 10) where dirty data refers to invalid material that includes expressions and special strings that cannot be analyzed independently. In addition, we directly deleted users with dirty data instead of excluding individual posts that had problematic strings while preserving and analyzing all usable posts, because when we did so, the users could not meet other criteria. Their posts were obtained from the official Sina microblog API.

Based on the sex and age characteristics of people who died by suicide, termed the suicide group, we randomly selected people from the entire platform who had never suffered from suicidal ideation, referred to as the non-suicide group. Because all of the posts of the suicide group were before the year 2013, apart from the aforementioned criteria, additional inclusion criteria for the non-suicide group were as follows: (1) the posts published before 2013 do not contain any suicide keywords; (2) no more than 1000 original posts were published before 2013. According to these criteria, 15 microbloggers’ posts that were published before 2013 were downloaded from the official Sina microblog API. Ethical approval was obtained from the Institute of Psychology, the Chinese Academy of Sciences.

### 2.2. Data Analysis

First, five postgraduates with expertise in analyzing suicidal posts were trained to annotate microbloggers’ posts based on RST (Mann and Thompson, 1988). The inter-rater reliability reached 0.83 after three rounds of training. Then, the SPSS 22 software package was used for the computational statistical analysis. The frequency of rhetorical relations was summarized using descriptive statistics. To compare group differences, a chi-square test was used.

## 3. Results

According to the information they showed on their homepages and some media coverage, the suicide group consisted of 4 males and 11 females. The average age for them was 21.71 years (SD = 5.34 years) with 8 missing data. The group of excluded social media users with suicidal risk comprised 14 males and 16 females, and the average age for them was 22.55 years (SD = 5.38 years). There was a significant gender difference between the two groups ($\chi^{2}$ = 30.48, *df* = 1, *p* < 0.001. Meanwhile, there was no significant difference between the two groups in age (*t* = −0.34, *df* = 25, *p* = 0.74). For the non-suicide group, there were also 4 males and 11 females. The average age for them was 22.20 years (SD = 3.39 years) with no missing data. There was no significant difference between the two groups in age (*t* = −0.24, *df* = 20, *p* = 0.81).

For the suicide group, there were 10,111 posts in total, comprising 437,814 words. On the other hand, for the non-suicide group, there were 3740 posts in total, which consisted of 197,081 words. We also analyzed and classified the rhetorical relations of those posts. For the suicide group, there were 17,997 rhetorical relations in total, which accounted for 4.11% of their total corpus. For the non-suicide group, there were 3785 rhetorical relations in total, which accounted for 1.92% of their total corpus. There was significant difference between the two groups in the number of posts (*t* = 2.16, *df* = 16.44, *p* = 0.046) and rhetorical relations (*t* = 3.18, *df* = 15.18, *p* = 0.006), whereas there was no significant difference between the two groups regarding the number of words (*t* = 1.55, *df* = 24.11, *p* = 0.134). The average score and standard deviation of the numbers of posts, words, and rhetorical relations for the two groups are shown in Table 2.

To eliminate the effect of the different word quantities of the two groups in analyzing rhetorical relations, we counted all kinds of rhetorical relations per 100,000 words. That is, we divided the rhetorical relation count by the word count to obtain the number of rhetorical relations contained per 100,000 words. The results are summarized in Table 3. As can be observed, there were similarities and differences between the two groups at the same time. For example, the highest frequency of rhetorical relation in both groups was Joint, which was observed to be 82.01% within the suicide group and 56.23% within the non-suicide group. Moreover, the two groups did not use Motivation, Preparation, Non-volitional cause, Non-volitional result, Volitional cause, Volitional result, or Multi-nuclear restatement. Meanwhile, the non-suicide group also did not use Antithesis, Justify, Evaluation, Means, Otherwise and Unless. After controlling for word quantity, the differences in the total number of rhetorical relations between the suicide (M = 274.03, SD = 258.16) and non-suicide (M = 117.94, SD = 30.45) groups became marginally significant (*t* = 1.99, *df* = 19.60, *p* = 0.06). Other than Concession, Evidence, Justify, Evaluation, Otherwise, and Joint, the differences between the two groups were significant among other the rhetorical relations, as shown in Table 3.

## 4. Discussion

Under the framework of RST, this study aimed to analyze social media users’ online posts. We compared the rhetorical structures of the posts between microbloggers who had died by suicide and microbloggers without suicidal ideation. Our findings demonstrated that when compared against Chinese social media users who had no suicidal ideation, people who had died by suicide used significantly more rhetorical relations (*t* = 3.18, *df* = 15.18, *p* = 0.006). This phenomenon became marginally significant after controlling for word quantity.

Interestingly, compared to social media users without suicidal ideations, social media users who died by suicide not only posted more frequently, but also used more rhetorical relations in their posts. Existing research has pointed out that people at risk of suicide often suffer from emotional distress [19]. Moreover, stigma is one of the important factors that hinders people with suicide risk in seeking help [20]. By inference, people with suicide risk who are afraid of stigma in the real world but are going through anguish would perceive safer opportunity to express their misery and seek help online. Therefore, they would submit a higher number of posts than people who are not suffering from suicidal ideation. RST has demonstrated that the effect of rhetorical relations is to make discourse an effective and understandable tool for human communication [21]. Moreover, more rhetorical relations represent more sophisticated semantic relations [11]. One possible reason for people who died by suicide using more rhetorical relations in their posts is that the feelings and cognitions they wish to verbalize are more complex. Therefore, they require more rhetorical relations to make the text smooth and coherent. At the same time, the number of words between the two group was not significantly different. One possible reason for this finding is that Sina Weibo has a word limit of 140 Chinese characters, which restricts the length of the microblog.

Moreover, other than Concession, Evidence, Justify, Evaluation, Otherwise, and Joint, the differences between the two groups were significant among other rhetorical relations. Social media uses who died by suicide used were more likely to use Antithesis, Background, Enablement, Restatement, Summary, Circumstance, Condition, Interpretation, Means, Purpose, Solutionhood, Unconditional, Unless, Contrast, List and Sequence than those with no suicidal ideation. Meanwhile, social media users with no suicidal ideation did not use Antithesis, Justify, Evaluation, Means, Otherwise and Unless at all. Concession, Evidence and Justify belong to a presentational basis that is aimed to increase positive regard, belief, and acceptance [13]. These results showed that both Chinese social media users with and without suicidal risk do not have a higher level to increase positive regard, belief, or acceptance by using those three kinds of rhetorical relations. Evaluation and Otherwise belong to a basis that is designed to express parts of the subject matter of the text [13]. These results showed that there is no significant difference between the two groups in terms of using Evaluation and Otherwise in their online posts. Joint represents the lack of a rhetorical relation between the nuclei. Both groups used Joint most frequently, which may demonstrate the writing style of Chinese social media users. Their posts were often multinuclear, and no relation was claimed to hold between the nuclei [13].

The non-suicide group also did not use Antithesis, Justify, Evaluation, Means, Otherwise and Unless. Among them, the differences of Antithesis, Means and Unless were significant. The effect of Antithesis is that the writer favors one idea and not the other, which can increase readers’ positive regard for the nuclei. The effect of Means is to make readers recognize that the method or instrument in the satellite tends to make the realization of nuclei more likely. The effect of Unless is to let readers understand that nuclei are realized if satellite is not realized. Social media users who died by suicide frequently talked about their suicide plans online to form suicide pacts [22,23]. Moreover, when people have suicidal ideation, they become hesitant and contradictory, they do not want to live, but neither do they want to die [24]. This could be the reason for social media users who died by suicide using significantly more Antithesis, Means and Unless than social media users without suicide risk do not.

There exists a similarity between the two groups, too. Neither group used Motivation, Preparation, Non-volitional cause, Non-volitional result, Volitional cause, Volitional result, or Multi-nuclear restatement. Rimrott pointed out that Motivation typically appears in advertisements to encourage consumers to buy goods or services [25]. Because we chose personal Sina Weibo accounts instead of profitable subscriptions, the users were not engaged in selling anything to others. Therefore, they rarely used Motivation in their posts. At the same time, because Weibo is mainly used to record trifles of life or to express ones’ own emotions or opinions, there is no room for Preparation, which is often used in expository texts [26]. Non-volitional cause, Non-volitional result, Volitional cause, Volitional result, and Multi-nuclear restatement are all rhetorical relations talking about causal relationship with different focuses. For example, the effect of Non-volitional cause is to make readers recognize satellites as causes of nuclei when the nuclei are not volitional actions. The results showed that Chinese social media users seldom used causal relationships in their posts. Multi-nuclear restatements have multiple nuclei contained in one unit. Microbloggers did not use them, possibly due to the word limits of posts. In contrast to the results of a previous study based on posts and comments in Reddit finding that the most frequently used rhetorical relation was Elaboration [12], the most frequently used rhetorical relation for our study in both groups was Joint. Joint represents the lack of a rhetorical relation between nuclei, which means people talked about irrelevant and independent topics within a single post. Therefore, our findings make a theoretical contribution to the field by providing rhetorical relation comparisons between people who died by suicide and people without suicide risk in the Chinese social media user population. To our knowledge, this is the first study to focus on the identification of rhetorical relation of social media users who died by suicide in China.

Although this study makes several contributions to the existing field of research, there are several limitations to this work. First, because this research is largely exploratory and not hypothesis driven, caution should be taken in generalizing our findings. More research is urgently required in this field in the future. Second, as a tentative study, even though there were abundant posts for analysis, the number of participants was relatively small, and they were mainly unmarried young females with college degrees, which is similar to a previous study focused on Twitter states. That is, the study contained mainly women aged 15–29 years [27]. It is worth noting that there was a significant gender difference between social media users who died by suicide in this study and those who were deleted. Further analyses would be necessary to generalize these findings to other populations of social media users with different demographic characteristics and suicidal situations. Third, in this work, the annotations were completed by trained college students. It has been demonstrated that different annotators of RST can achieve a high degree of consistency, whether they annotate different discourses or the same discourse, because RST uses a unified definition for all types of discourse [14]. Using automatic analytical tools could not only raise efficiency, but also allow the possibility to extend the scope of research. Today, there are free rhetorical annotation tools online [28]. Therefore, future studies could build machine learning models based on RST and use them alongside automatic analytical tools. Fourth, the main purpose of this study is to introduce RST to suicide post analysis. No other methods were used. Based on the results of this study, more analytical methods could be combined to work together in the context of textual analysis. For example, content analysis and rhetorical relations can be combined to gain a better understanding of texts. Finally, some results of our study are different from the findings obtained by studies based on Western culture. A previous publication demonstrated that Chinese abstracts prefer to use Evaluation and Elaboration, whereas English abstracts are inclined to use more Evidence [28]. In addition, different genres have substantial impacts on the use of rhetorical relations [11]. This work needs to be replicated in different cultures to determine the influence of cultural characteristics and expression customs on rhetorical relations. For example, future research could compare the similarities and differences in the rhetorical relations used in posts on Facebook, Reddit, and Sina Weibo.

## 5. Conclusions

This study aimed to investigate the rhetorical relations of online posts in Chinese social media users who died by suicide. Our findings demonstrated that the difference between social media users who died by suicide and those without suicidal ideation were significant among most rhetorical relations. These preliminary findings show that rhetorical relations can be used as discriminants to distinguish social media users who died by suicide from those who do not have suicidal ideations. This study expands RST to a Chinese population as well as into the context of online textual analysis and suicidal rhetorical relations, which are all innovative steps. Therefore, this work paves the way for future research to focus on RST and serve in the identification of social media users who died by suicide, which could subsequently contribute to the development of culturally tailored and system-focused suicide prevention and intervention strategies.

## Figures and Tables

**Table 1:** Effects of the thirty rhetorical relations.

Rhetorical Relations	Effect
Antithesis	Increasing readers’ positive regard towards the nucleus
Background	Increasing readers’ ability to comprehend the nucleus
Concession	Increasing readers’ positive regard towards the nucleus
Enablement	Increasing readers’ potential ability to perform action in the nucleus
Evidence	Increasing readers’ belief of the nucleus
Justify	Increasing readers’ readiness to accept the writer’s right to present the nucleus
Motivation	Increasing readers’ desire to perform action in the nucleus
Preparation	Increasing readers’ readiness or interests to read the nucleus
Restatement	Helping readers recognize that the satellite is a restatement of the nucleus
Summary	Helping readers recognize that the satellite is a shorter restatement of the nucleus
Circumstance	Helping readers recognize that the satellite provides the framework for interpreting the nucleus
Condition	Helping readers recognize that the realization of the nucleus depends on the realization of the satellite
Elaboration	Helping readers recognize that the satellite provides additional detail for the nucleus
Evaluation	Helping readers recognize that the satellite assesses the nucleus
Interpretation	Helping readers recognize that the satellite relates the nucleus to a framework of ideas not involved in the knowledge presented in the nucleus itself
Means	Helping readers recognize that the method or instrument in the satellite makes the realization of the nucleus more likely
Non-volitional cause	Helping readers recognize that the satellite is a cause of the nucleus
Non-volitional result	Helping readers recognize that the nucleus could have caused the situation in the satellite
Otherwise	Helping readers recognize that the dependency relation of prevention between the realization of the nucleus and the realization of the satellite
Purpose	Helping readers recognize that the activity in the nucleus is initiated in order to realize the satellite
Solutionhood	Helping readers recognize that the nucleus is a solution to the problem presented in the satellite
Unconditional	Helping readers recognize that the nucleus does not depend on the satellite
Unless	Helping readers recognize that the nucleus is realized provided that the satellite is not realized
Volitional cause	Helping readers recognize that the satellite is a cause for the volitional action in the nucleus
Volitional result	Helping readers recognize that the nucleus could have caused the situation in the satellite
Contrast	Helping readers recognize the comparability and the difference yielded by the comparison is being made
Joint	None
List	Helping readers recognize the comparability of linked items
Multi-nuclear restatement	Helping readers recognize the re-expression by the linked items
Sequence	Helping readers recognize the succession relationships among the nuclei

**Table 2:** The numbers of posts, words, and rhetorical relations of participants by suicidal status.

	Post Count (M ± SD)	Word Count (M ± SD)	Rhetorical Relation Count (M ± SD)
Suicide group	674.07 ± 729.28	29,187.60 ± 33,567.356	1199.80 ± 1130.24
Non-suicide group	249.33 ± 216.23	13,138.73 ± 21,926.73	252.33 ± 232.43

**Table 3:** Rhetorical relation proportions of participants by suicidal status.

Rhetorical Relations	Suicide Group (M ± SD)	Non-Suicide Group (M ± SD)	$\chi^{2}$	*p*
Antithesis	0%	0.19%	7.07	0.017 *
Background	2.27%	3.60%	21.23	0.014 *
Concession	0.11%	0.32%	10.71	0.067
Enablement	0.19%	0.48%	12.60	0.011 *
Evidence	0.11%	0.39%	5.77	0.651
Justify	0%	0.04%	-	1
Restatement	2.05%	2.56%	27.04	0.000 ***
Summary	0.32%	2.74%	21.92	0.002 **
Circumstance	0.56%	2.86%	21.69	0.002 **
Condition	0.46%	1.38%	24.35	0.000 ***
Elaboration	4.98%	6.40%	27.75	0.049 *
Evaluation	0%	0.16%	-	1
Interpretation	0.40%	3.07%	18.80	0.005 **
Means	0%	0.11%	5.64	0.042 *
Otherwise	0%	0.04%	2.01	0.483
Purpose	0.19%	0.48%	18.88	0.000 ***
Solutionhood	1.28%	1.41%	19.25	0.032 *
Unconditional	0.16%	0.27%	19.13	0.001 **
Unless	0%	0.13%	6.97	0.017 *
Contrast	1.39%	3.36%	26.79	0.003 *
Joint	82.01%	56.23%	28.03	1
List	1.17%	1.75%	19.16	0.002 **
Sequence	2.85%	12.03%	24.27	0.004 **

* *p* < 0.05; ** *p* < 0.01; *** *p* < 0.001.

## Data Availability

The data presented in this study are available on request from the corresponding author. These data are not publicly available in the interest of ethics.
